# Rab-GDI Complex Dissociation Factor Expressed through Translational Frameshifting in Filamentous Ascomycetes

**DOI:** 10.1371/journal.pone.0073772

**Published:** 2013-09-19

**Authors:** Fabienne Malagnac, Céline Fabret, Magali Prigent, Jean-Pierre Rousset, Olivier Namy, Philippe Silar

**Affiliations:** 1 Univ Paris Sud, Institut de Génétique et Microbiologie, UMR8621, Orsay, France; 2 Univ Paris Diderot, Sorbonne Paris Cité, Institut des Energies de Demain (IED), Paris, France; 3 Centre national de la recherche scientifique, Institut de Génétique et Microbiologie, UMR8621, Orsay, France; University of Wisconsin – Madison, United States of America

## Abstract

In the model fungus *Podospora anserina*, the *PaYIP3* gene encoding the orthologue of the *Saccharomyces cerevisiae* YIP3 Rab-GDI complex dissociation factor expresses two polypeptides, one of which, the long form, is produced through a programmed translation frameshift. Inactivation of *PaYIP3* results in slightly delayed growth associated with modification in repartition of fruiting body on the thallus, along with reduced ascospore production on wood. Long and short forms of PaYIP3 are expressed in the mycelium, while only the short form appears expressed in the maturing fruiting body (perithecium). The frameshift has been conserved over the evolution of the *Pezizomycotina*, lasting for over 400 million years, suggesting that it has an important role in the wild.

## Introduction

All living cells have to accurately convert gene information into proteins in a complex multistep process. Translation is suspected to be the key step where errors may occur. One of the main issues is to keep translating the correct reading frame from the start codon to the stop codon. The reading frame is defined by the start codon, as the genetic code is unpunctuated; any shift leads to an aberrant protein. Spontaneous frameshift errors occur at low levels in comparison to missense errors. The ribosome has developed several mechanisms to allow proper translocation of the tRNAs in the different sites. Signals are present on some mRNAs and induce an alternative reading of the genetic code by diverting the standard rules. These elements are very efficient at disrupting ribosome accuracy, increasing error rate from background (<5×10^−5^) to 50% [1]. Such programmed events are known as recoding. Among the recoding sites identified, programmed frameshifting (PRF) are the most frequently found signals. +1 frameshifting motifs are widespread and regulate several important cellular functions in both eukaryotes and prokaryotes [2], [3] whereas −1 frameshifting events are currently mainly limited to viral genomes [4], with very few exceptions such as *dnaX* in prokaryotes or the *edr*/*PEG10* and *Ma3* genes in eukaryotes [5], [6], [7], [8]. −1 frameshifting motifs are minimally formed by a slippery sequence of the general structure X XXY YYZ (the initial reading frame is indicated) where tRNAs shift reading frame, and a stimulatory element (mainly a secondary structure) [9], [10] located 5–9 nucleotides (nt) downstream of the slippery sequence.

In the filamentous fungus *Podospora anserina*, it was hypothesized about 30 years ago that translation accuracy changes during cell differentiation allowing the production of key regulatory proteins required for the development of this model fungus [11]. Indeed, tampering with accuracy of the *P. anserina* cytosolic translation apparatus results in impaired development [12], [13]. Especially, decreasing the error rate triggers abnormal ascospore formation [14], [15] and the development of an epigenetic cell degeneration mechanism known as Crippled Growth [16]. The regulatory proteins, whose diminished expression could be responsible for both alterations as hypothesized by Picard-Bennoun, are still unknown. Moreover, altering translation accuracy results in altered lifespan [17], [18], through presently unknown mechanism(s) [15], [17], [19], [20]. While sequencing the *P. anserina* genome, we identified potential candidates regulated by recoding that could explain the importance of maintaining a certain level of translational error [21]. Among these candidate genes, a −1 frameshifting site located in the *P. anserina* orthologue of the *Saccharomyces cerevisiae* YIP3/PRA1 protein appears conserved in *Pezizomycotina*. YIP3 is a Rab-GDI displacement factor involved in endoplasmic reticulum to Golgi transport. It is conserved in animals [22] and plants [23]. We present here a functional analysis of this factor in *P. anserina* showing that its production is indeed regulated by a programmed translational −1 frameshift, which appears widely conserved in filamentous ascomycetes.

## Materials and Methods

### Fungal strains and culture conditions

All the *P. anserina* strains used in this study derived from the “S” (Uppercase S) wild-type strain that was used for sequencing [21], [24]. The latest genome sequence and EST derived from the S strain are available at http://podospora.igmors.u-psud.fr. Construction of the *Δmus51::su8–1* strain lacking the mus-51 subunit of the complex involved in end-joining of broken DNA fragments was described previously [25]. DNA integration in this strain proceeds almost exclusively by homologous recombination. Standard culture conditions, media and genetic methods for *P. anserina* have been described [26] and the most recent protocols can be accessed at http://podospora.igmors.u-psud.fr/more.php; the M2 minimal medium is a medium in which carbon is supplied as dextrin, and nitrogen as urea. The methods used for nucleic acid extraction and manipulation have been described [27], [28]. Transformation of *P. anserina* protoplasts was carried out as described previously [29].

For the frameshifting measurement, the FY1619 *S. cerevisiae* strain was used (MATa ura3–52 trp1Δ63 his3Δ200 leu2Δ1).

### Frameshifting efficiency

The reporter plasmid for frameshifting measurement was the pAC vector [30]. The *Podospora* target sequences, corresponding to either the −1 frameshift sequence (Podo-1) (5′-GCTTTTTCCGGGGAGGTGGTCTAGGTGGTCGGCCACGAGCTCCCAAATTTTCGCCCCAT-3′) or the in-frame control sequence (Podo0) (obtained by addition of a G at the 5′ end) were inserted at the *Msc*I restriction site located at the junction between β-galactosidase and the firefly luciferase coding sequences. Hybrid sequences consisting of the IBV (Infectious Bronchitis Virus) slippery site with either the IBV spacer sequence (IBV-Podo-1 sp6) (5′-TATTTAAACGGGTACGGGAGGTGGTC**A**AGGTGGTCGGCCAC**GAG**CTCCCAAATTTTCGCCCC-3′) or the Podospora spacer sequence (IBV-Podo-1 sp2) (5′-TATTTAAACCGGGGAGGTGGTC**T**AGGTGGTCGGCCAC**GAG**CTCCCAAATTTTCGCCCCA-3′) followed by the Podospora pseudoknot were also cloned into the pAC *MscI* site, as well as a sequence consisting of the IBV slippery site out-of frame with the IBV spacer sequence and the *P. anserina* pseudoknot (IBV-Podo0) (5′- TTATTTAAACGGGTACGGGAGGTGGTC**A**AGGTGGTCGGCCAC**GAG**CTCCCAAATTTTCGCCCCAT-3′). The β-galactosidase and firefly luciferase activities were quantified in the same crude extract as described previously [31] for standard growth conditions. Quantifications were the mean of six independent measurements. The efficiency, defined as the ratio of firefly luciferase activity to β-galactosidase activity, is expressed as percentage, and calculated by dividing the firefly luciferase/β-galactosidase ratio obtained from the −1 frameshift construct by the same ratio obtained with the in-frame control construct [30]. Statistical significance was determined using the Mann–Whitney test (XL STAT 2007 software).

### Deletion of *PaYIP3*

*PaYIP3* was inactivated by replacing the *PaYIP3* CDS with a hygromycin B-resistance marker. The flanking regions of the *PaYIP3* gene were amplified by PCR, using the primers 8470GF (5′- aagcttccggatccgagaataacc-3′) and 8470GR (5′-tctagacggtttggagaaggaaaagg-3′) for the upstream sequence, and 8470DF (5′-ctcgagaccattaacgcccgttgttt-3′) and 8470DR (5′-aagcttcttcgcgaccttctcaa-3′) for the downstream sequence. Two primers contain sites for restriction enzymes to help in cloning. The PCR products were digested with *Xba*I/*Hind*III for the upstream region and with *Hind*III/*Xho*I for the downstream region and cloned into the pBC-hygro vector [13] digested with *Xba*I and *Xho*I. The plasmid recovered was then linearized with *Hind*III and introduced into the *P. anserina Δmus51::su8–1* strain by transformation. Numerous transformants were obtained. Selected transformants were crossed with the wild-type strain to remove the *Δmus51::su8–1* marker and obtain *mat+* and *mat−* strains carrying the deletion. Southern Blot analysis confirmed that for two transformants the *PaYIP3* gene was correctly deleted (Figure S1). One such mutant was selected for further phenotypic analyses.

### Complementation of PaYIP3 and creation of truncated and corrected CDS (coding sequence)

To complement the *PaYIP3^Δ^* mutant, the wild-type gene was amplified by PCR using the Phusion® High-Fidelity DNA Polymerase (Fermentas), *P. anserina* wild-type genomic DNA and primers 8470DF and 8470GR. The product was cloned into the pBC-phleo vector [13], to yield pPaYIP3^+^. Sequencing of the insert proved that no mutation occurred in *PaYIP3* during amplification and cloning. The pPaYIP3^+^ plasmid was then introduced by transformation into the *PaYIP3^Δ^* mutant. Twenty-six transformants, all with a wild-type phenotype, were recovered. Two were selected for further studies and crossed with wild-type. The phleomycin-resistant *PaYIP3^Δ^* F1 progeny carrying the *PaYIP3^+^* transgene had a wild-type phenotype, demonstrating that restoration of the phenotype was due to the introduction of a wild-type copy of the *PaYIP3* gene.

The corrected version of the PaYIP3 CDS was PCR amplified using the Phusion® High-Fidelity DNA Polymerase, *P. anserina PaYIP3^C^-GFP* genomic DNA (see below) and primers 8470DF and 8470RL (5′-agcggccgcttaactgacacaaacgacaatcg-3′). Because the *PaYIP3^C^* allele was amplified from a GFP-tagged version it has no stop codon. Therefore the PCR product was cloned into the pBC-phleo vector [13] along with the Rib2 terminator harboring a TAA codon in its 5 prime end, to yield pPaYIP3^C^. Sequencing the insert proved that no mutation occurred in *PaYIP3^C^* during amplification and cloning. The pPaYIP3^C^ plasmid was then introduced by transformation into the *PaYIP3^Δ^* mutant. 19 transformants, all with a *PaYIP3^Δ^* mutant phenotype, were recovered. Two were selected for further studies and crossed with wild-type. Analysis was performed on F1 progeny with the *PaYIP3^C^ PaYIP3^Δ^* genotype.

The truncated version of PaYIP3 CDS was PCR amplified using the Phusion® High-Fidelity DNA Polymerase, *P. anserina* wild-type genomic DNA and primers 8470DF and 8470RC (5′-atctagactagaccacctccccggaaaaagcc-3′). The PCR product was cloned into the pBC-phleo vector [13], along with the Rib2 terminator to generate the *PaYIP3^T^* plasmid. Sequencing the insert proved that no mutation occurred in *PaYIP3^T^* during amplification and cloning. The pPaYIP3^T^ plasmid was then introduced by transformation into the *PaYIP3^Δ^* mutant. Thirteen transformants, showing a range of phenotype intermediates between the wild-type and the *PaYIP3^Δ^* mutant, were recovered. Three of them were selected for further studies and crossed with wild-type. Analysis was performed on the F1 progeny with the *PaYIP3^T^ PaYIP3^Δ^* genotype.

### Plasmid constructions for GFP-tagging

To construct a C-terminal GFP fusion of the frameshifted protein, the 535 bp 3′-end fragment of the 3′ ORF of the *PaYIP3* gene was PCR-amplified using the orf3′w-bgl2 (5′-GAAGATCTGGCTCTAGTGCATCCAGGACAC-3′) and orf3′c-sma1 (5′-TTTCCCGGGTCGCTTTCCTTCTAGCAGTACC-3′) oligonucleotides, containing, respectively, the *Bgl*II and *Sma*I sites (underlined in the sequence). After enzymatic digestion, the fragment was cloned into the *Bgl*II and *Sma*I sites of the peGFP-hygro vector, resulting in the pYIP3^+^-GFP vector. peGFP-hygro contains the hygromycin B resistance gene from pBC-hygro inserted into the *Not*I site of the peGPF-1 vector from Clontech.

A C-terminal GFP fusion with the frameshifted protein was artificially obtained using the pYIP3^C^-GFP vector, which was constructed by cloning a 2 kb fragment of the PaYIP3 gene, in which the frameshift was eliminated. To this end, two PCR-fragments corresponding to the 5′ ORF (860 bp) and the 5′-end of the 3′ ORF (1208 bp) were amplified, respectively, with the orf5′w-sal1 (5′-ACGTGTCGACCGGTTCAGCCGTTCTTTCGGAGC-3′) (*Sal*I site underlined) and phase-c (5′-CCTCCCC*GGAAAAA*GCC**C**TCATCGATAGGCTTG-3′) oligonucleotides, and with the phase-w (5′-CAAGCCTATCGATGA**G**GGC*TTTTTCC*GGGGAGG-3′) and orf3′c-sma1 oligonucleotides. Phase-w and phase-c oligonucleotides are complementary and contain the slippery sequence (indicated in italic) of the −1 frameshifting site. They also carry a supplemental nucleotide (in bold) compared to the genomic sequence in order to place the 3′ ORF in frame with the 5′ ORF. Both fragments were PCR-joined, *Sal*I- and *Sma*I-digested and cloned into the correspondingly digested peGFP-hygro vector to yield the pYIP3^C^-GFP vector.

A last C-terminal GFP fusion was constructed with only the 5′ ORF. A 869 bp fragment of the the 5′ ORF was PCR-amplified using the orf5′w-sal1 and the orf5′c-sma1 (5′-AGACCCGGGCCACCTCCCCGGAAAAAGCCTC-3′) (*Sma*I site underlined) oligonucleotides. After digestion with both the *Sal*I and *Sma*I enzymes, and cloning into the *Sal*I-*Sma*I-digested peGFP-hygro, the pYIP3^T^-GFP vector was obtained.

All constructs were verified by sequencing and then transformed into the *Δmus51::su8-1* strain. DNA was extracted from selected transformants, PCR-amplified so as to sequence the inserted chimaeric genes. For each of the three plasmids (pYIP3^+^-GFP, pYIP3^C^-GFP and pYIP3^T^-GFP), two transformants, in which a correct integration of the chimaeric GFP transgenes had occurred, were selected and crossed with wild-type. In the progeny, *mat+* and *mat−* strains carrying the GFP chimaeric genes and devoid of the *Δmus51::su8-1* were selected for observation. GFP localization was identical in the two transformants of all three constructs.

### Western blot analysis

The anti-GFP monoclonal antibodies from Roche Applied Science (catalog number 11814460001) were used for the detection of GFP-fusion proteins by western blot analysis. Proteins were extracted as described [32].

### Microscopy analysis

Pictures were taken with a Leica DMIRE 2 microscope coupled with a 10-MHz Cool SNAP_HQ_ charge-coupled device camera (Roper Instruments). They were analyzed with ImageJ. The GFP filter was the GFP-3035B from Semrock (Excitation: 472 nm/30, dichroïc: 495 nm, Emission: 520 nm/35).

### Phylogenetic analysis

The trees were constructed using PhyML [33] with the default parameters [34] and 100 bootstrapped data sets. Trees were visualized with the iTOL server [35].

## Results

### The structure of the -1 translational frameshifting motif of *PaYIP3*

**Figure 1:**
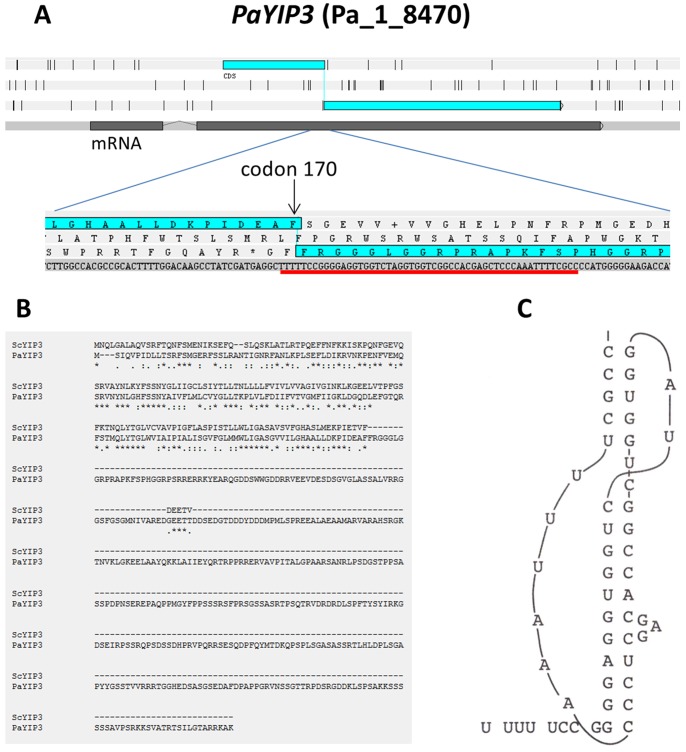
Structure of the *PaYIP3* gene and translational −1 frameshift. (**A**) Overall structure of the *PaYIP3* gene and enlargement of the translation −1 frameshift site. (**B**) Comparison of the *S. cerevisiae* YIP3 protein (ScYIP3) with PaYIP3. (**C**) Possible pseudoknot folding of the sequence underlined in **A**.

As seen in Figure 1A, the mRNA of the *P. anserina PaYIP3* gene (CDS number Pa_1_8470) overlaps two open reading frames (ORFs). The small one located at the 5′ end of the messenger is similar to the *S. cerevisiae YIP3* gene (YNL044W), while the larger one at the 3′ end is not present in this yeast (Fig. 1B). This second ORF has no known functional domain and is only found in the genomes of *Pezizomycotina*, a large group of *Ascomycota* fungi (see phylogenetic analysis below). Translation from the first 5′ ORF should produce a 19.5 kDa protein similar to YIP3. It is possible to generate a larger protein of 61.6 kDa in *P. anserina* that would encompass both the small 5′ and large 3′ ORFs by hypothesizing a -1 frameshift. Analysis of 12 independent cDNAs obtained during the *P. anserina genome* sequencing project showed that the mRNA sequence was identical to that of the gene except for a 173 bp intron located in the 5′ untranslated region [21]. This indicates that the putative frameshift is not corrected by either RNA editing or splicing and thus it had to occur during translation. We could not find a canonical frameshift signal; however, examination of the *PaYIP3* sequence suggested that frameshifting could happen at codon n° 170, since there is at this position the sequence “U UUU UCC”, a potential slippery sequence [10]. Despite the fact that this slippery sequence does not match the consensus X XXY YYZ motif, this sequence would still allow tRNA tandem slippage by pairing of the tRNA^Ser^ (IGA) to the -1 codon (UUC) since G-U base pairing between codon and anticodon is possible. Moreover, there is a sequence capable of forming a stem-loop and possibly a pseudoknot two nt downstream of the potential slippery sequence (Fig. 1C), a feature that should increase frameshifting frequency [9], [10]. Such close vicinity between the slippery sequence and the stimulatory element is rather unusual for a −1 frameshifting stimulatory element. However the G-C stretch at the base of stem 1 is reminiscent of the very well-studied IBV pseudoknot [36]. Despite these unusual features we were able to detect the long frameshifted protein fused with GFP, demonstrating that this −1 frameshifting motif is indeed functional (see below).

### The -1 frameshift of PaYIP3 has been conserved during the evolution of Pezizomycotina

**Figure 2:**
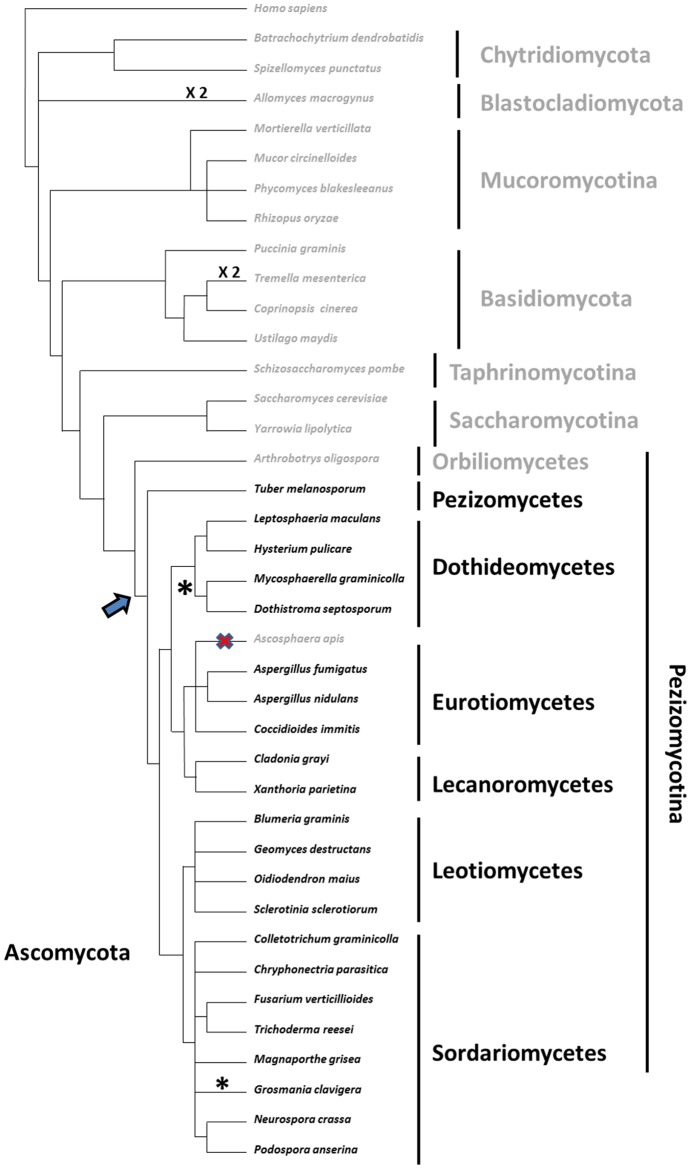
Evolution of YIP3 in fungi. The presence (species in black) and absence of the 3′ ORFs (species in grey) of YIP3 was mapped on the most probable phylogenetic tree of the Eumycota [43], [46]. The most parsimonious scenario of evolution is a single appearance of the 3′ ORF after the split of the Orbiliomycetes from the other Pezizomycotina (slanted arrow) and a secondary loss in *A. apis* (cross). * denotes the two groups for which a+1 frameshift is hypothesized and ×2 the species in which a duplication of YIP3 has occurred.

**Figure 3:**
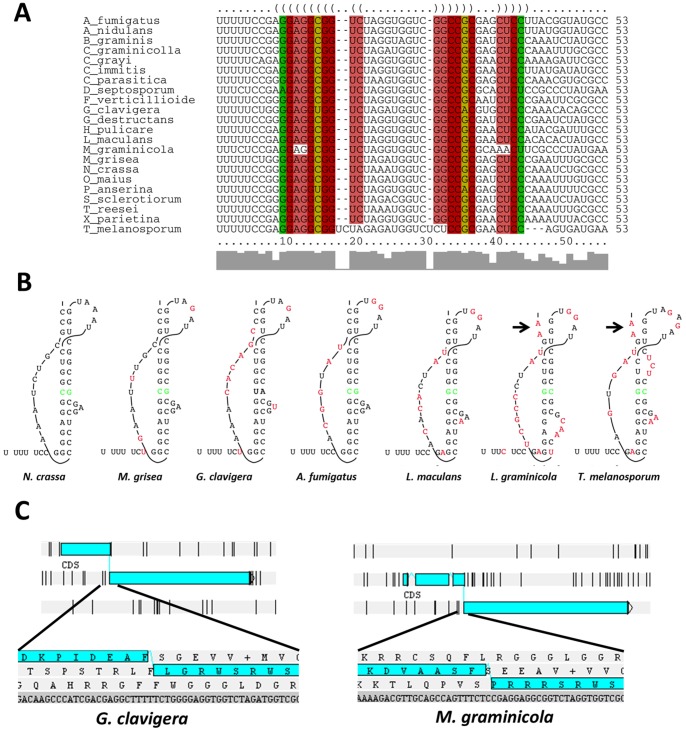
Conservation of the frameshift site. (**A**) Alignment of the YIP3 frameshift site in selected fungi using LocARNA [47] to identify conserved secondary structures. Compatible base pairs are colored according to the number of different types C-G, G-C, A-U, U-A, G-U or U-G of compatible base pairs in the corresponding columns. In this way, the hue shows sequence conservation of the base pair. The saturation decreases with the number of incompatible base pairs indicating structural conservation of the base pair. (**B**) Manual prediction of pseudoknot structures present after the frameshift site in selected species. Conservative changes compared to the *P. anserina* structures are in green and those in non-conservative changes in red. Arrows point to unpaired bases preventing knot tethering in *M. graminicola* and *T. melanosporum*. (**C**) Structure of the frameshift site in *G. clavigera* and *M. graminicola* showing that the downstream ORF is in the +1 frame.

In our previous study [21], we observed that the YIP3 -1 frameshifting motif is conserved during evolution, at least in *Pezizomycotina*. The availability of additional fungal genome sequences now makes it possible to refine the phylogenetic analysis. To this end, the YIP3 orthologues from representative species, whose genome sequences were available in public databases and covering the entire diversity of the *Eumycota*, were manually annotated. The sequences of the 5′ short and 3′ large ORFs of the *Pezizomycotina* proteins, along with those of other fungi, were aligned and phylogenetic trees were constructed (Figure S2 and S3). Both trees were compatible with the known evolution of the *Eumycota* and *Pezizomycotina* (Fig. 2). In most fungi, YIP3 is encoded by a single gene (Fig. 2). The presence of a C-terminal extension was detected in all species of *Pezizomycotina*, except for *Ascosphaera apis* and *Arthrobotrys oligospora* (Fig. 2). Examination of the structure downstream the frameshift sites showed that it was highly conserved in most *Pezizomycotina* and compensatory changes could be detected in the pseudoknot structure (Fig. 3A and 3B). Otherwise, changes were restricted to the predicted loops (Fig. 3B). However, in the case of *Tuber melanosporum*, the adopted structure may not be a pseudoknot, but rather a simple stem-loop. Interestingly, in two clades, the *Capnodiales* (eight species investigated) and the *Ophiostomatales* (one species investigated), the C-terminal extension was not in the −1 frame but in the +1 frame (Fig. 2 and Fig. 3C). The UUUUU slippery sequence and the pseudoknot were conserved in *Grosmania clavigera*, the *Ophiostomatales*, while the UUUUU sequence was changed to UUUCU and only a small stem loop could be predicted in the case of *Mycosphaerella graminicola*, a *Capnodiales* (Fig. 3C). This could indicate that a single tRNA slippage would occur in this sequence instead of the classical tandem tRNA slippage.

### Deletion of *PaYIP3* uncovers a growth phenotype but evidences no role in ascospore formation and Crippled Growth development

**Figure 4:**
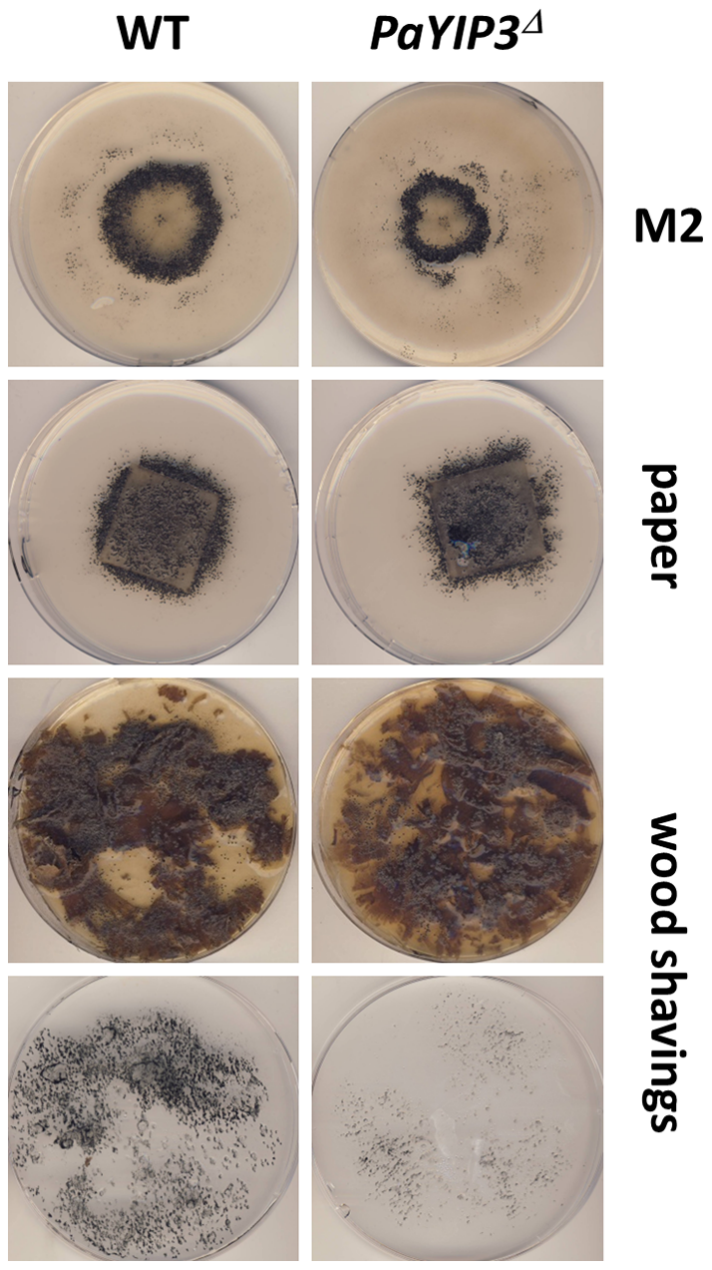
Phenotypes of the *PaYIP3* *^Δ^* mutant. Heterokaryotic *mat+/mat−* strains of the indicated genotypes were inoculated at the center of the plate and incubated at 27°C. Pictures were taken 10 days later. For wood shaving medium, the plates and their covers, onto which ascospores were projected forming the black material, were photographed.

To define the role of *PaYIP3* in the physiology of *P. anserina*, the gene was inactivated by replacing its entire coding sequence by a hygromycin B resistance marker, *i.e.*, the deleted gene is unable to produce either the short or the long forms of PaYIP3 (see Materials and Methods). The *PaYIP3^Δ^* mutant strain was investigated during the entire life cycle of the fungus and no difference was detected during the reproductive phase, i.e., kinetics and modalities of fruiting body development, ascospore genesis and germination. On the contrary, mycelium defects appeared. First, the mycelium of *PaYIP3^Δ^* grew slightly more slowly than wild-type (6+0.2 mm/d instead of 7+0.2 mm/d). However, growth resumed at the same speed as wild-type after 3 to 4 days of incubation. This was accompanied by an altered pattern in the repartition of the fruiting bodies (perithecia; Fig. 4) when grown on M2 minimal medium. Indeed, on this medium the wild-type produced perithecia mainly as a 1 cm-thick ring with an internal diameter of 1 cm, whereas in the mutant the ring was 0.7 cm thick and its internal diameter was 0.7 cm. Moreover, although the kinetics of perithecium development was identical after fertilization, perithecia from *mat+*/*mat−* heterokaryotic mutant cultures yielded ascospores half a day before wild-type cultures, suggesting that fertilization occurred earlier in the mutant, in line with the smaller diameter of the ring of fruiting bodies. Finally, when grown on a medium with wood shavings as sole carbon source, fertility of the mutant was diminished and fewer ascospores were produced, while growth with Whatman paper (i.e. pure cellulose) as carbon source was only marginally modified (Fig. 4). Significantly, Crippled Growth and life span were not modified in the mutant (data not shown).

### Complementation of the *PaYIP3^Δ^* mutant

**Figure 5:**
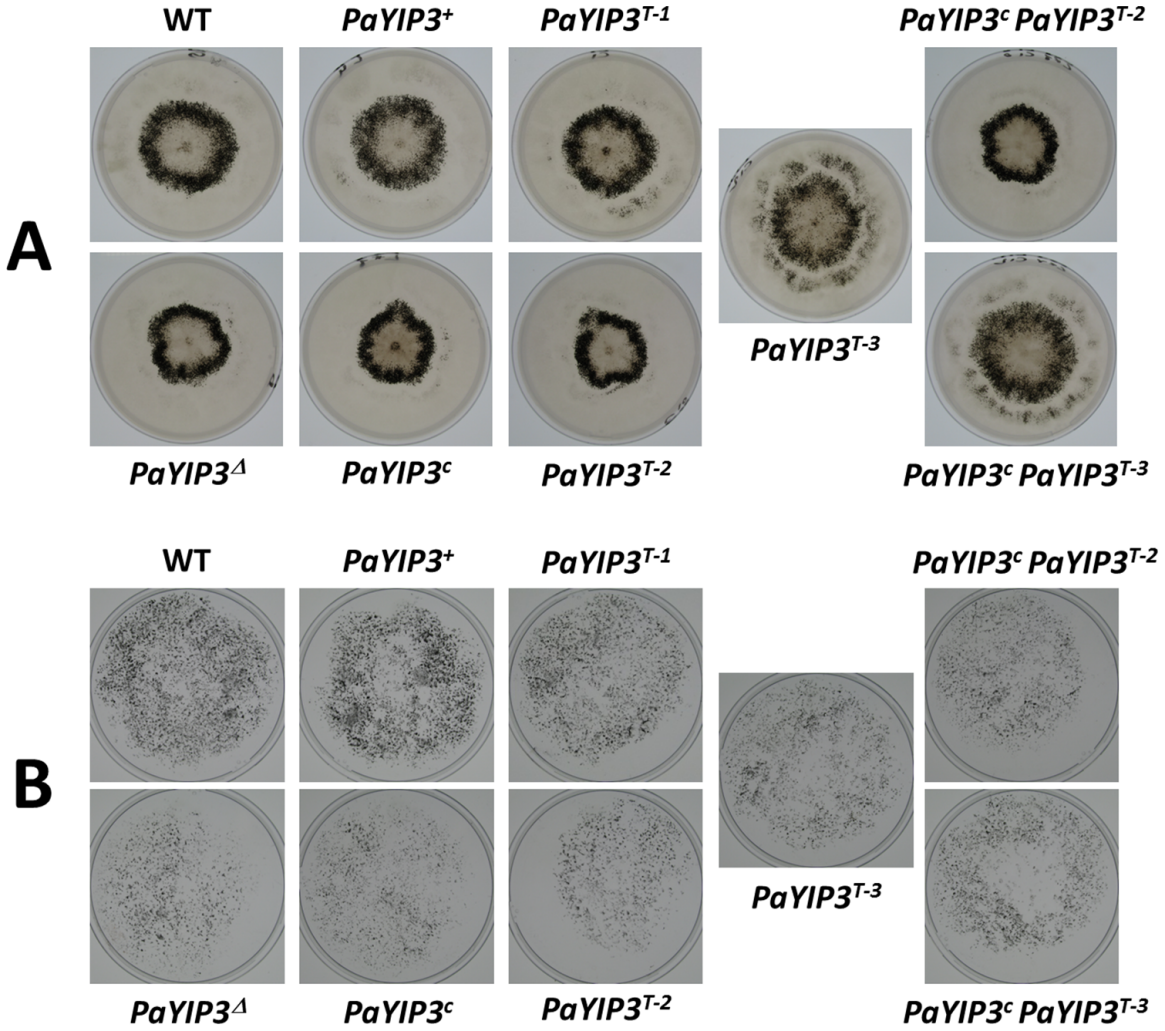
Complementation of the *PaYIP3* *^Δ^* **mutant.** Heterokaryotic *mat+/mat−* strains of the indicated genotypes were treated as those in Figure 4. The three independent transformants of PaYIP3^T^ are representatives of the observed phenotypic variation. (**A**) Ring formation on M2 medium; (**B**) ascospore production on wood shaving medium. Only the plate covers are shown.

To assess whether the ring and wood shaving phenotypes were due to deletion of *PaYIP3*, a wild-type copy of the *PaYIP3* gene was introduced by transformation into the *PaYIP3^Δ^* mutant. As seen in Figure 5, complete restoration of the wild-type phenotype was observed in transformants carrying an ectopic copy of *PaYIP3^+^*, whereas the complemented strains formed a wild-type ring of perithecia and produced as much ascospores as wild-type on wood shaving medium, demonstrating that inactivation of *PaYIP3* was responsible for all the phenotypes observed (compare *PaYIP3^Δ^* with *PaYIP3^+^*).

The mutant was also transformed with truncated and corrected alleles of the *PaYIP3* gene. The *PaYIP3^T^* truncated allele was obtained by removing the 3′ ORF and produced only the 19.5 kDa polypeptide. The *PaYIP3^c^* corrected allele was created by inserting a C in codon n°168, which is just upstream of the frameshift site, and produced the 61.6 kDa polypeptide resulting from the fusion of the proteins produced by the 3′ and 5′ ORFs. Introduction of *PaYIP3^c^* did not result in the restoration of a wild-type phenotype: ring and ascospore production on wood shaving medium was identical to that observed with the *PaYIP3^Δ^* mutant (Fig. 5). A heterogeneous restoration depending on the transformants was observed with the *PaYIP3^T^* allele. Recovery ranged from complete (*e.g.*, the T1 transformant had a wild-type phenotype) to inexistent (*e.g.*, the T2 transformant has a *PaYIP3^Δ^* mutant phenotype, Fig. 5), while others presented a modified repartition of perithecia and diminished fertility on wood shaving medium (*e.g.*, the T3 transformant had a more diffuse ring and produced reduced amount of ascospores on wood shaving medium). Integrative transformation in *P. anserina* results mostly in non-homologous insertion. Variability in the complementation observed with the truncated allele could be explained by influence of the insertion point on the expression of the transgene. To check whether expression of both the truncated and corrected form in the same strain could rescue a complete wild-type phenotype, we constructed by genetic crosses strains carrying both the corrected and truncated alleles of *PaYIP3*. As seen in Figure 5, these strains exhibited the same phenotypes as those expressing the truncated allele alone, indicating that co-expression of both forms is not sufficient to recover a wild-type phenotype.

### Tagging the wild-type, short and long forms of PaYIP3 with GFP

**Figure 6:**
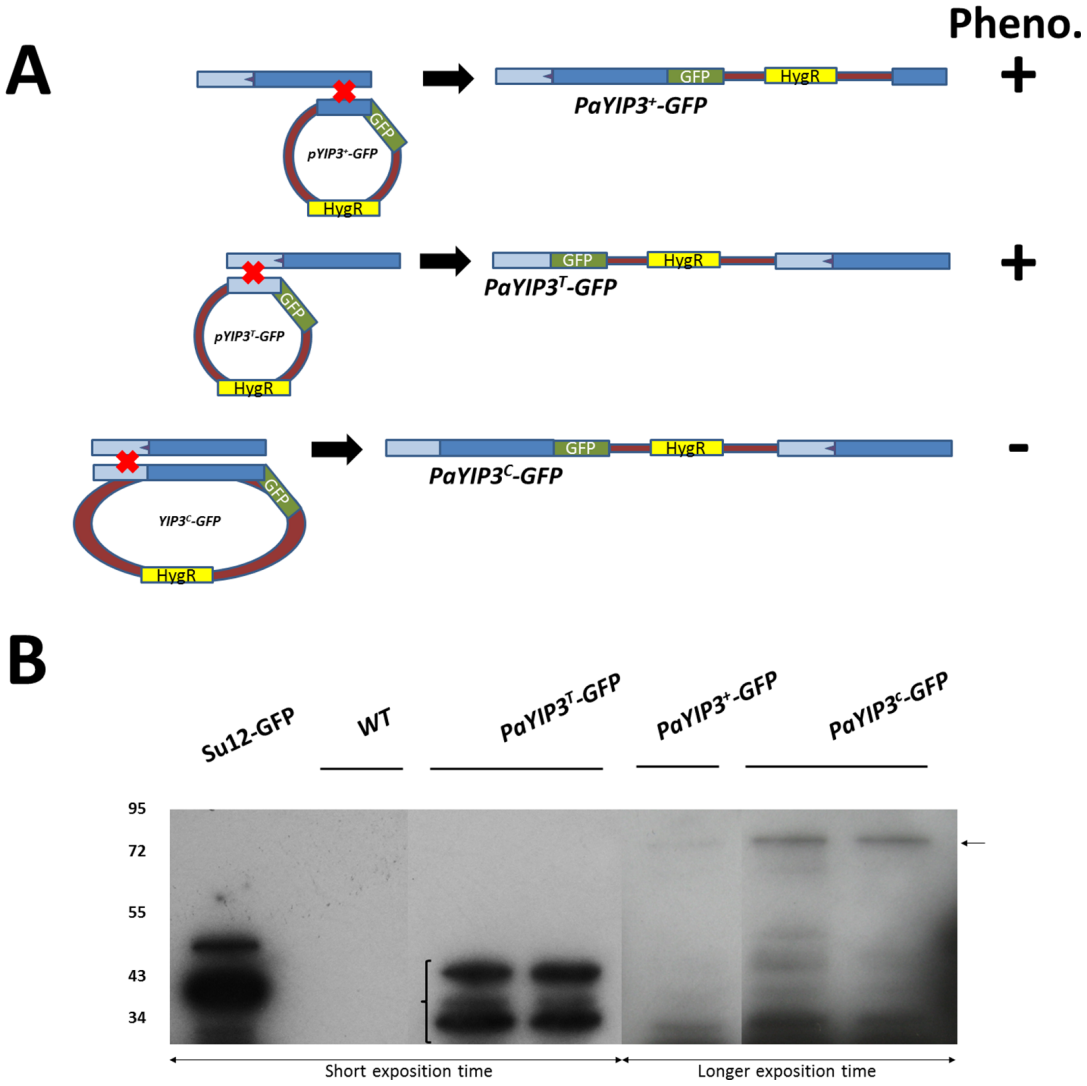
Western blotting analysis of chimaeric proteins. (**A**) Schematic representation of the integration events leading to the generation of the *PaYIP3^+^-GFP*, *PaYIP3^T^-GFP* and *PaYIP3^C^-GFP* chimaeric genes. In the case of *PaYIP3^T^-GFP* and *PaYIP3^C^-GFP* a complete copy of the CDS is still present in the genome downstream of the tagged gene. The strains carrying the *PaYIP3^T^-GFP* gene have a wild type phenotype, while those carrying the *PaYIP3^C^-GFP* gene have the *PaYIP3^Δ^* mutant phenotype (Figure 7). (**B**) Cellular extracts from transformants with the indicated genotypes were separated on a 10% acrylamide 1 mm-thick gel and probed with an anti-GFP antibody. The left part results from a short exposition time that reveals the control and the PaYIP3^T^ peptides. The right part is a longer exposition time to reveal the proteins produced from *PaYIP3^+^* and *PaYIP3^C^* transgenes. Brackets and arrow, respectively, indicate the short and the longer proteins specifically revealed by the anri-GFP antibody. Controls: 59 kDa fusion protein of Su12 ribosomal protein in frame with GFP (Su12-GFP, [48]).

To determine the *in vivo* expression level and localization of the short and long forms of YIP3, we constructed plasmids carrying chimaeric versions of the wild-type, truncated and corrected alleles by adding in frame the eGFP CDS at the 3′ end of *PaYIP3* (see Materials and Methods). Transformation of the three plasmids into *P. anserina* resulted in their integrations at the *PaYIP3* chromosomal locus by a single crossing-over, generating the modified loci depicted in Fig. 6A. In the case of the wild-type *PaYIP3^+^-GFP* transgene only a small ∼500 pb truncated PaYIP3 CDS was present downstream of the chimaeric gene, while for the two other constructs a complete CDS was retained. However, these copies had a 5′-untranslated region truncated just before the intron (Fig. 1), and thus lacked the promoter region as well as 350 pb of the 5′-UTR of the *PaYIP3* mRNA.

To ensure that the recovered GFP-transgenes were expressed, we monitored by Western blotting with an anti-GFP antibody the production of the proteins from *PaYIP3^+^-GFP*, *PaYIP3^T^-GFP* and *PaYIP3^C^-GFP* (Fig. 6B). A doublet of bands, likely due to different levels of posttranslational modification observed for proteins with reticulum/Golgi localization, was detected at ∼45 kDa for *PaYIP3^T^-GFP*, as well as a band of about 88 kDa for *PaYIP3^+^-GFP* and *PaYIP3^C^-GFP*. These sizes are expected for the fusion proteins, showing that they were actually produced in *P. anserina*. Although present in low amounts, the detection of a band of high molecular weight with the *PaYIP3^+^-GFP* construct demonstrated that the 61.6 kDa-long form of the protein is naturally produced by the wild-type allele.

**Figure 7:**
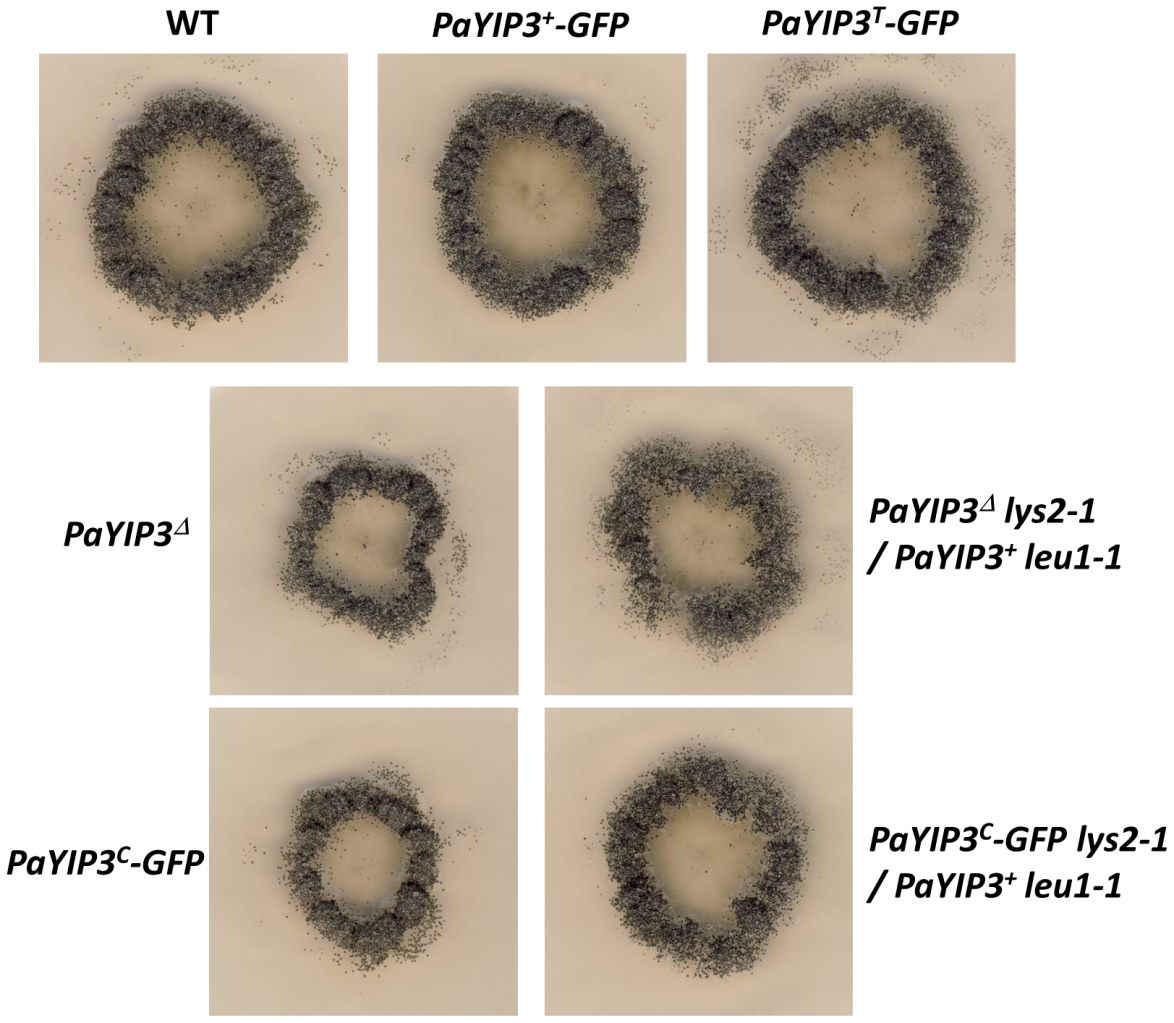
YIP3-GFP phenotypes and recessivity. Heterokaryotic *mat+/mat−* strains of the indicated genotypes were treated as those grown on M2 medium in Figure 4.

When analyzed on M2, the strains carrying the *PaYIP3^+^-GFP* and *PaYIP3^T^-GFP* transgenes grew and presented a ring of perithecia as did the wild-type strain, while those carrying *PaYIP3^C^-GFP* were similar to the *PaYIP3^Δ^* mutant (Fig. 7). To test whether the phenotype of *PaYIP3^C^-GFP* resulted from lack of activity of the remaining downstream wild-type CDS or to a dominant negative effect of the *PaYIP3^C^-GFP* copy, we constructed by genetic crossings balanced heterokaryon with the following genotype: *PaYIP3^C^-GFP lys2-1/PaYIP3^+^ leu1-1*. These had a wild-type phenotype (Fig. 7), showing that the *PaYIP3^C^-GFP* phenotype was recessive. The same expected result was observed with a *PaYIP3^Δ^-GFP lys2-1/PaYIP3^+^ leu1-1* heterokaryon used as control. Therefore, the PaYIP3^C^-GFP protein displayed no dominant negative effect. Hence as observed for the ectopic PaYIP3^C^ protein, PaYIP3^C^-GFP is not functional and the downstream copy of the gene is actually inactive despite having the complete *PaYIP3* CDS.

**Figure 8:**
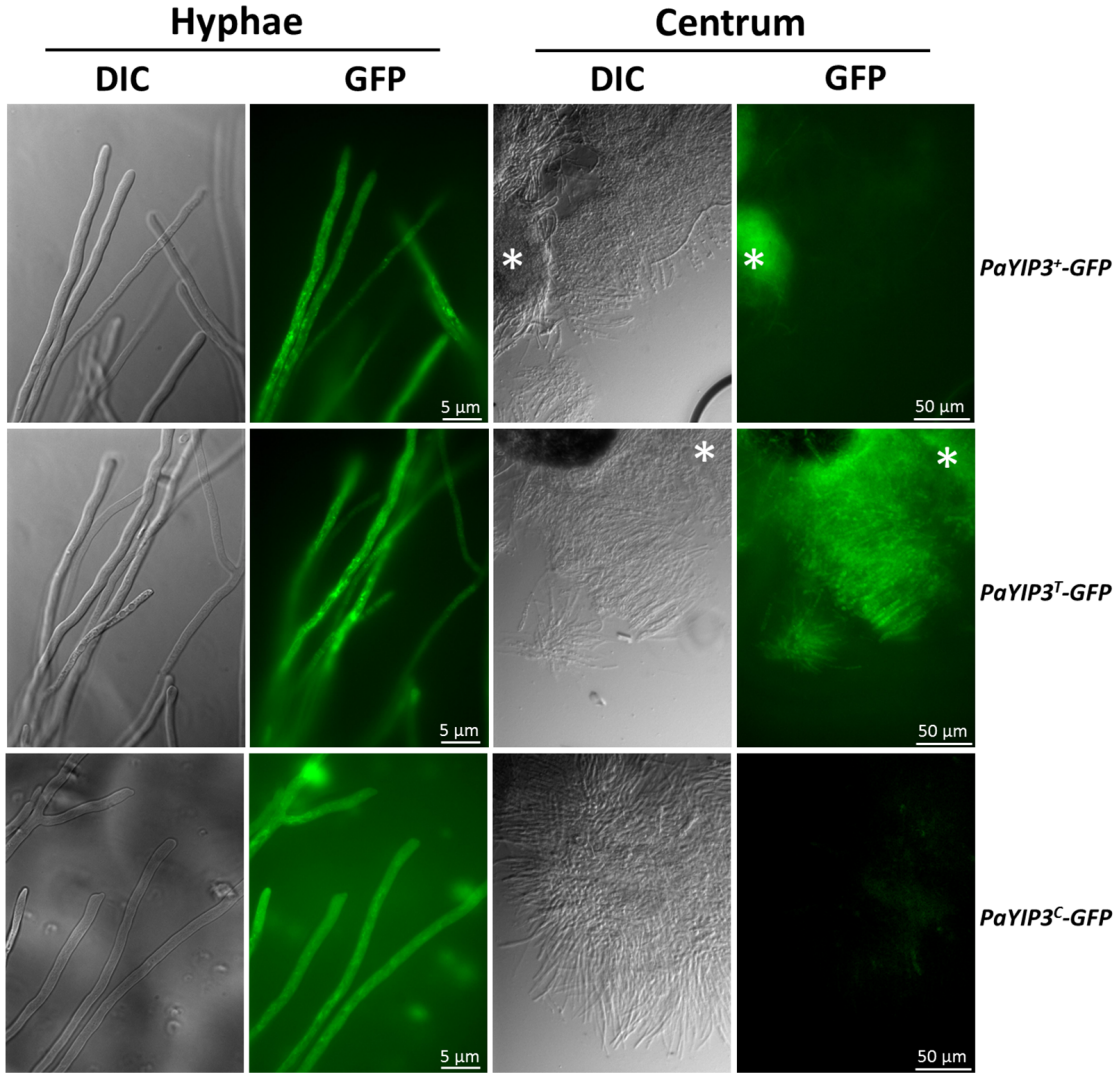
*In vivo* expression of *PaYIP3.* GFP expression in the indicated strains was assayed in hyphae and in the inner portion (centrum) of the fruiting bodies. * denotes hyphae included for comparison.

Microscopic examination revealed a clear expression of the wild-type fusion PaYIP3^+^-GFP protein in the hyphae confirming that the recoded form is efficiently expressed *in vivo* in the host. This longer protein seems to display the same localization as the smaller form (PaYIP^T^-GFP; Fig. 8). The corrected in-frame form (PaYIP3^C^-GFP) is more difficult to observe in the hyphae, as expected if it is present in lower amounts. In all three strains, the fluorescence was concentrated in foci compatible with a reticulum/Golgi localization as observed for the orthologous YIP3/PRA1/PRA2 protein [37], [38]. Intriguingly, while we did not detect fluorescence in the centrum of fruiting bodies from the *PaYIP3^+^-GFP* and *PaYIP3^C^-GFP* strains, a clear staining was observed with the *PaYIP3^T^-GFP* protein, suggesting that only this short form of the protein is present in this tissue.

### Quantification of -1 frameshifting efficiency

Unfortunately it is not possible to evaluate frameshifting efficiency by western-blot as the small peptide is not tagged with GFP in the PaYIP3^+^-GFP construct. Moreover western-blot results from PaYIP3^C^-GFP suggest that the longer form could be unstable, due to the weak intensity of the band (Fig. 6), which corresponds to 100% of corrected protein. To determine if the identified *PaYIP3* recoding sequence was able to promote −1 frameshifting, it was inserted between the β-galactosidase and firefly luciferase open reading frames of a yeast *S. cerevisiae* reporter plasmid [30]. The −1 frameshifting efficiency was quantified as described in Materials and Methods. The level was estimated to be 1%, which in *S. cerevisiae* indicated a low but true -1 frameshift event. We also replaced the slippery sequence by the very well characterized slippery sequence from the IBV −1 frameshift (T TTA AAC) in frame or out-of-frame. Positioning the IBV slippery sequence out-of-frame results in a significant drop in frameshifting efficiency (0.3%), indicating that the 1% obtained with the PaYIP3 sequence is significant. However, despite having tested several spacer distances between the IBV slippery sequence and the *PaYIP3* pseudoknot, it is clear that this pseudoknot is unable to stimulate frameshifting in the IBV sequence to the same extent as does the IBV pseudoknot does (Figure S4). This confirms the unusual nature of the frameshifting event that occurs on this sequence.

## Discussion

We present evidence that the *P. anserina* gene orthologous to *S. cerevisiae YIP3*, *PaYIP3*, is expressed in a complex fashion, whereby two proteins are produced with a single mRNA through a translational -1 frameshifting event. The mRNA produced by this gene encompasses two ORFs, the 5′ ORF is highly conserved and is similar to the CDS of *YIP3*, while the 3′ ORF is less conserved and present only in *Pezizomycotina*. The two ORFs can be expressed in *P. anserina* as a single polypeptide if a −1 frameshift occurs during translation. We were able to reveal *in vivo* a fusion protein corresponding to the translation of the two ORFs. We know from the analysis of EST that the mRNA does not undergo any modification (splicing, editing); therefore, only a translational event can explain the fusion protein. Several translational recoding events such as ribosome hopping or frameshifting could explain how the fusion protein is synthesized. However, two characteristic features reminiscent of frameshifting motifs are found in the overlapping sequence between the two ORFs: i) a pseudoknot that is an mRNA structure commonly stimulating −1 frameshifting [9], [10]; ii) a slippery sequence. This sequence does not match the −1 frameshifting slippery sequence consensus; nevertheless, the slippage of the tRNA^Ser^ (IGA), which base pairs to the UCC codon in the initial frame, can occur on the -1 codon (UUC) since G-U base pairing between codon and anticodon is possible. Another unconventional feature of this new frameshifting site is the proximity of the slippery sequence to the stimulatory pseudoknot. Indeed the slippery sequence is located only 2 nt upstream of the pseudoknot whereas the spacer region is usually around 5 to 9 nt.

To gain further details about this new frameshifting site we tested this motif in the yeast *S. cerevisiae* with a dual reporter system. Frameshifting efficiency was estimated as 1%, which is low but significant since frameshifting efficiency dropped to as low as 0.3% when the reading frame in which the ribosome encounters this slippery sequence was changed. Moreover our western-blot results suggest that frameshifting efficiency could be higher in the natural host. To better characterize the frameshifting mechanism the slippery site was replaced by a canonical slippery sequence from the IBV pseudoknot. Despite efforts to position the IBV slippery sequence either at 6 nt from the pseudoknot (the size of the IBV spacer region), or at 2 nt (the size of the *PaYIP3* pseudoknot) the IBV frameshifting efficiency in yeast (10–15%) was not reached and only a moderate increase with a 2 nt spacer was observed. This indicates that the *PaYIP3* pseudoknot is unable to stimulate frameshifting as does the IBV pseudoknot with a 6 nt spacer. However we cannot rule out that the slight increase observed with the 2 nt spacer is significant. This tentatively suggests that the *PaYIP3* pseudoknot may slightly improve basal frameshifting of the IBV slippery sequence when positioned 2 nt downstream. It is always difficult to extrapolate the functional role from a predicted structure, as the pseudoknot can fold in an inactive form [39]. However One plausible explanation comes from the lack of conservation of the potential slippery sequence between the two organisms, suggesting that the frameshifting event does not occur by a dual tRNA slippage as for IBV but more probably by a single P-tRNA slippage, similarly to frameshifting found in bacterial transposable elements [40]. This would also explain why the *PaYIP3* pseudoknot is unable to fully stimulate dual tRNA slippage on the IBV sequence. This is also in accordance with the fact that this pseudoknot displays several unusual features. It is positioned very close to the slippery sequence (only 2 nt), and there is a highly conserved bulge of 3 nt in stem 1 of the pseudoknot. Such a 3 nt bulge has already been described for the Edr frameshifter pseudoknot [6] but remains very unusual. Mutagenesis of Edr frameshifting supports a tandem tRNA slippage model and the bulge found in Edr pseudoknot does not play any role in frameshifting. The situation could be very different for *PaYIP3* frameshifting as a trans-acting factor could bind this pseudoknot to stimulate frameshifting in the host. This frameshifting is not conserved in *S. cerevisiae* so this unknown factor would be absent in this species explaining the inability of the pseudoknot to stimulate frameshifting at the IBV slippery site. Whatever the mechanism, this frameshifting site is functional in *P. anserina*. It is worth mentioning that up to now the only two eukaryotic genes (*Ma3* and *Edr/PEG10*), that use a -1 frameshift to extend the size of a protein, are derived from domesticated retroviruses. No such signature is found in PaYIP3 suggesting that this will be the first cellular gene using a -1 frameshifting event to extend the protein length and not related to a retrovirus.

In *S. cerevisiae*, YIP3 facilitates the dissociation of endosomal Rab–GDI complexes [41]. However, YIP3 may have multiple functions in the cell, which are as yet not well characterized [37]. In *P. anserina*, deletion of *PaYIP3* triggers three phenotypes. Indeed, on M2 standard medium, the mutants initiate their growth slightly more slowly than wild-type and differentiate a smaller ring of fruiting bodies that produce ascospores half a day earlier. They are also impaired in their ability to produce abundant ascospores on medium with wood shavings as sole carbon source. At the present time, the molecular defects underlying these phenotypes are unknown, but defects in protein routing may account for them. In particular, degradation of wood shavings relies on the secretion of many enzymes that act synergistically, and impairment in secretion can alter the ability to degrade lignocellulose. Remarkably, the mutants are not affected in their ability to complete maturation of ascospores (although their yield is reduced) and to undergo crippled growth, the two main developmental phenotypes triggered by increasing translation accuracy. Likewise, their lifespan is not modified, as typically seen in accuracy mutants. Therefore, PaYIP3 is not the factor produced by a recoding event and hypothesized to regulate either one of these phenomena [11], [15], [16], [17]. Some of the other *P. anserina* genes found to be potentially regulated by recoding [21] could be involved.

The possible role of PaYIP3 frameshifting in *P. anserina* is not clear at the present time. Indeed, expression of the 19.5 kDa alone can restore a wild-type phenotype, when present at the *PaYIP3* chromosomal location (Fig. 5). However, when present at an ectopic location, complementation may not be complete, likely due to abnormal expression of the transgenes integrated at an ectopic location (Fig. 5), unlike what is observed for the wild-type allele with frameshift. Moreover, expression of the 61.6 kDa polypeptide alone is unable to restore a wild-type phenotype, indicating that it is inactive. It does not appear to act as a dominant negative form (Fig. 5). Finally, expressing both the 19.5 kDa and 61.6 kDa forms within the same cells from different transgenes does not improve phenotypic rescue. Several explanations can be proposed. Possibly, the laboratory conditions tested are not appropriate to detect an effect of the lack of frameshift. Redundancy from another factor along with PaYIP3 may be masking an effect of the frameshift product. The 19.5 kD and 61.6 kD forms may need to be present in defined ratio to observe an effect. Another possibility would be that doing an error during translation rather than producing two different polypeptides is important. This could allow for spatial or temporal regulation of PaYIP3. Interestingly, while expression of the 19.5 kDa form is detected in the perithecium centrum, expression of the 61.6 kDa form was not detected either from the wild-type allele or the corrected allele. Possibly, the C-terminus of the 61.6 kDa polypeptide acts as a degradation signal in the centrum. This situation is reminiscent of the *S. cerevisiae PDE2* gene. Indeed this gene undergoes translational stop codon readthrough producing a short and a long protein. The longer protein is destabilized and quickly degraded by the proteasome [42]. In the case of *PaYIP3* this degradation signal is tissue specific adding a supplementary layer of regulation. The reason for the lack of the 61.6 kDa in the centrum of fruiting bodies is not clear, as we detected no change in the ascospore maturation and ejection processes in the mutants. Possibly, a protein degradation mechanism exists in the centrum so as to ensure that only certain proteins are transmitted to the progeny. The 61.6 kDa protein may be recognized and specifically degraded, without causing any harmful effect.

Whatever the role of the recoding, it seems important from an evolutionary point of view as it is conserved in all the *Pezizomycotina* that we have investigated except for *A. oligospora* and *A. apis*. *A. apis* is a fungus that infects beehives and may present features associated with parasitism, such as simplified metabolism. More interesting is the fact that *A. oligospora* belongs to the *Orbiliomycetes*, a class of *Pezizomycotina*, which is believed to be the first to have diverged during evolution [43]. If correct, the most parsimonious explanation of the phylogenetic pattern of the C-terminal extension would be its origin after the split of the *Orbiliomycetes* from the other *Pezizomycotina* and its removal from the ancestors of *A. apis* due to their parasitic lifestyle (Fig. 7). This split likely occurred more than 400 million years ago [44]. During this time, the −1 frameshift was conserved except in two orders for which a+1 frameshift may occur. In the *Ophiostomatales*, the frameshift site is well conserved, *i.e.*, a slippery sequence and a pseudoknot are present, yet only a+1 frameshit can join the 5′ and 3′ ORFs. In the *Capnodiales*, the slippery sequence and the pseudoknot are not conserved, but the two ORFs are conserved in a+1 frameshift configuration. This confirms that recoding during translation is important for the regulation of YIP3 in filamentous ascomycetes. A recent report [45] shows that recodings are involved in metabolic control in a wide range of fungi through the production of distinct polypeptides in the targeting of enzymes to different compartments. Here, we show that a gene involved in routing proteins is also subjected to translation recoding control, possibly adding a level of complexity to the metabolic network.

## Supporting Information

Figure S1 Top: Alignement of selected fungal *YIP3* CDS with the 5′ORF of *YIP3* in various Pezizomycotina. Bottom: corresponding PhyML tree tested with 100 bootstraps.
(TIF)

Figure S2 Top: Alignement of selected 3′ORF of *YIP3* from various Pezizomycotina. Bottom: corresponding PhyML tree tested with 100 bootstraps.
(TIF)

Figure S3 **Southern blot analysis of the** ***PaYIP^Δ^*** **mutant.** Top: schematic representation of wild-type and deleted *PaYIP3* loci. Bottom: autoradiogram obtained after probing *BamH*I-digested DNA with the probe highlighted on top.
(TIF)

Figure S4 −**1 Frameshifting efficiency quantified in S.** ***cerevisiae*** **.** The −1 frameshifting efficiency of each Podo and IBV-Podo sequence was quantified as described in Materials and Methods. The *p*-value with the Podo-1 data is indicated for each sequence tested.
(TIF)
