# Supplementary figures and images for: Rab-GDI Complex Dissociation Factor Expressed through Translational Frameshifting in Filamentous Ascomycetes
Source: PLoS One. 2013 Sep 19;8(9):e73772. doi: 10.1371/journal.pone.0073772 (PMC3777964; doi:10.1371/journal.pone.0073772)

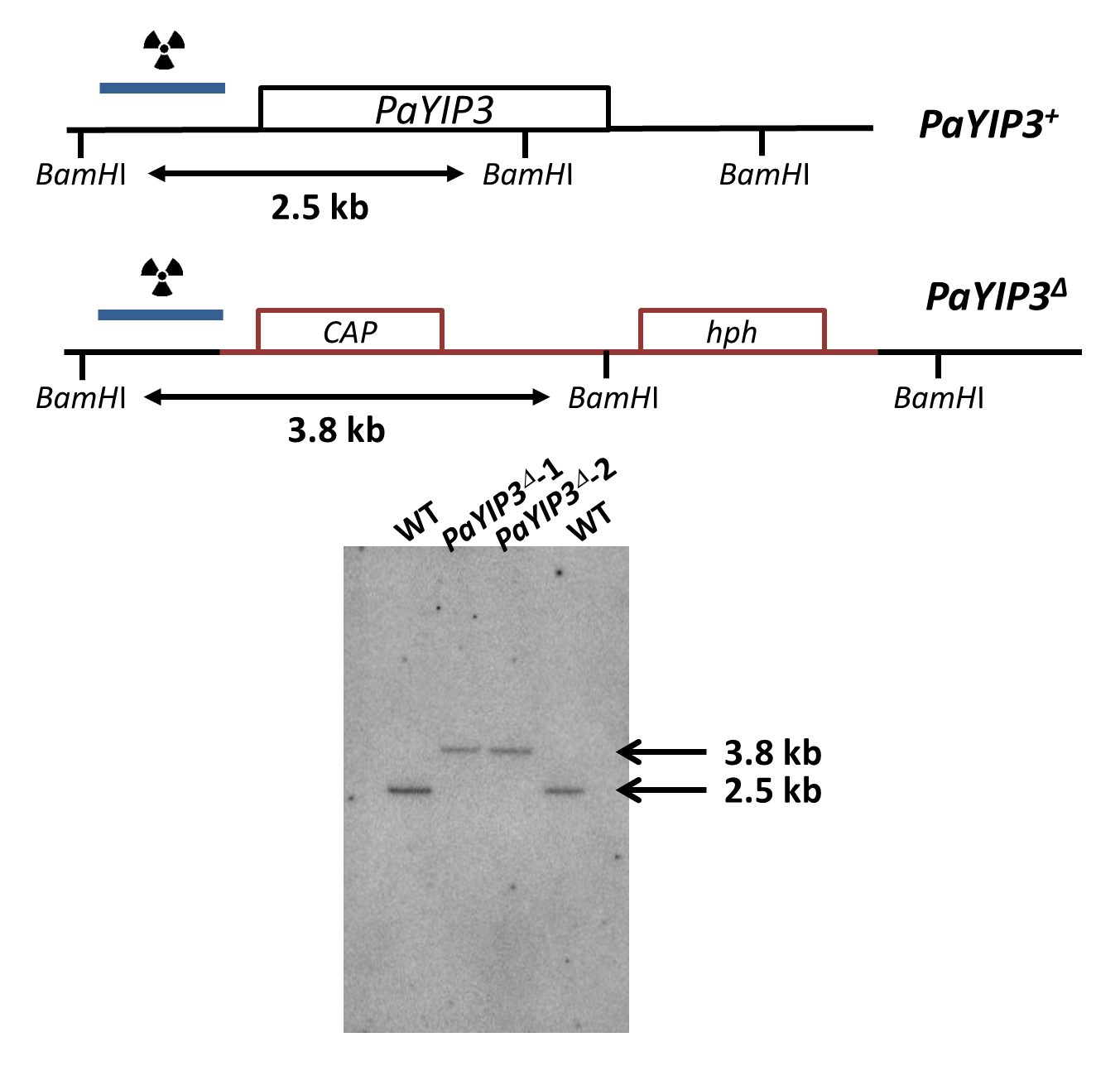

Supplement: Figure S1 — Top: Alignement of selected fungal YIP3 CDS with the 5′ORF of YIP3 in various Pezizomycotina. Bottom: corresponding PhyML tree tested with 100 bootstraps. (TIF) [file pone.0073772.s001.tif]

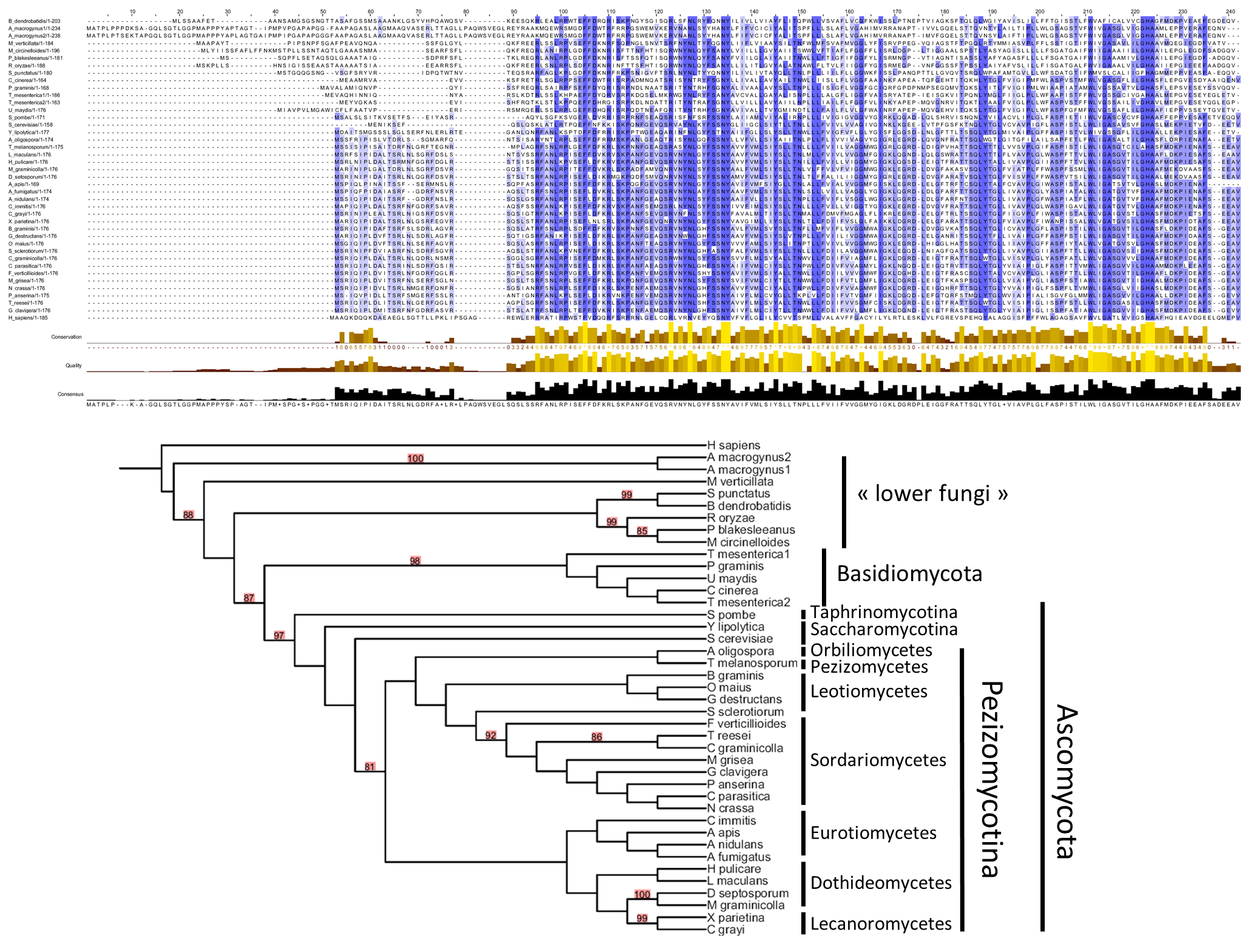

Supplement: Figure S2 — Top: Alignement of selected 3′ORF of YIP3 from various Pezizomycotina. Bottom: corresponding PhyML tree tested with 100 bootstraps. (TIF) [file pone.0073772.s002.tif]

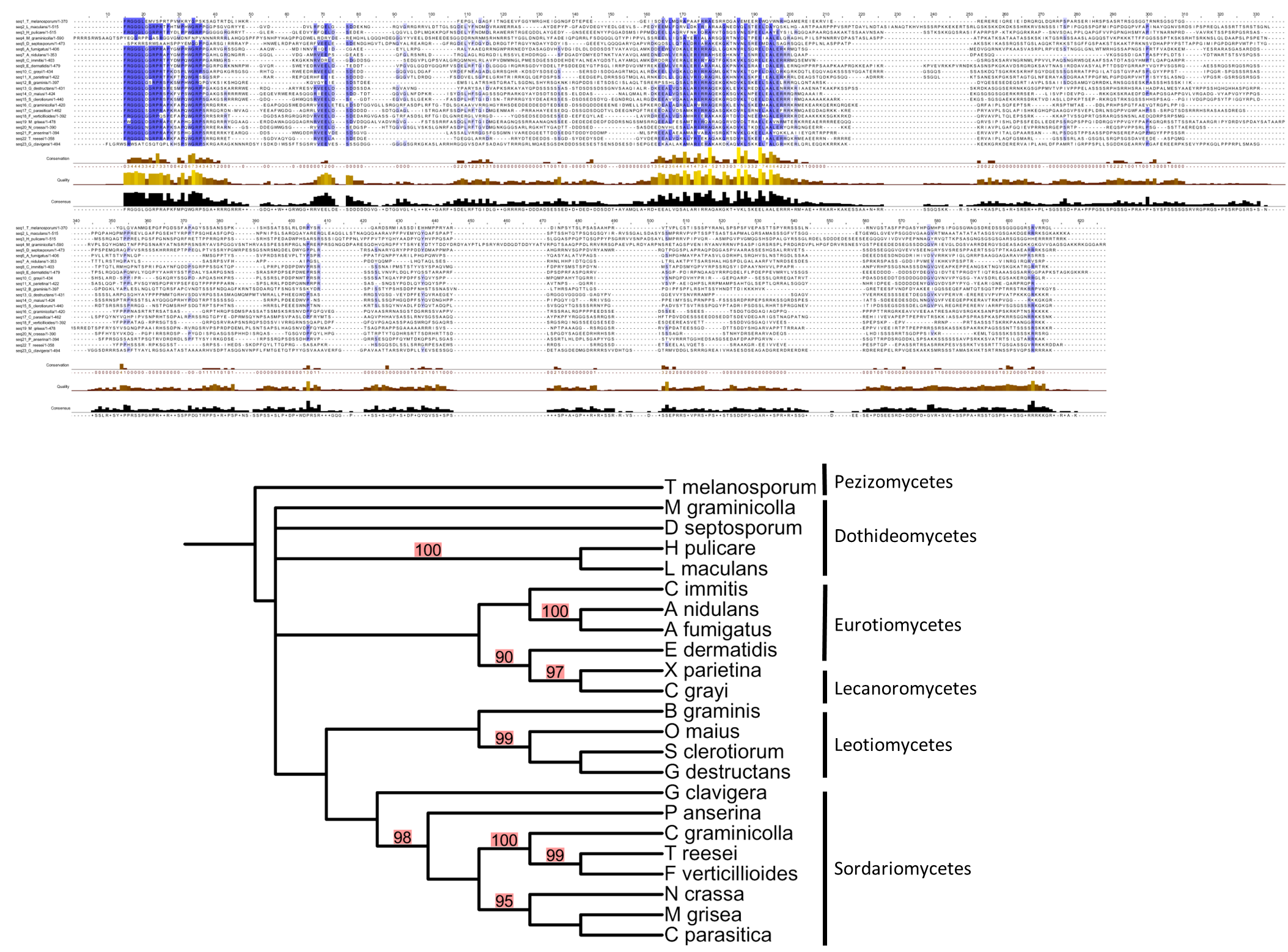

Supplement: Figure S3 — Southern blot analysis of the PaYIPΔ mutant. Top: schematic representation of wild-type and deleted PaYIP3 loci. Bottom: autoradiogram obtained after probing BamHI-digested DNA with the probe highlighted on top. (TIF) [file pone.0073772.s003.tif]

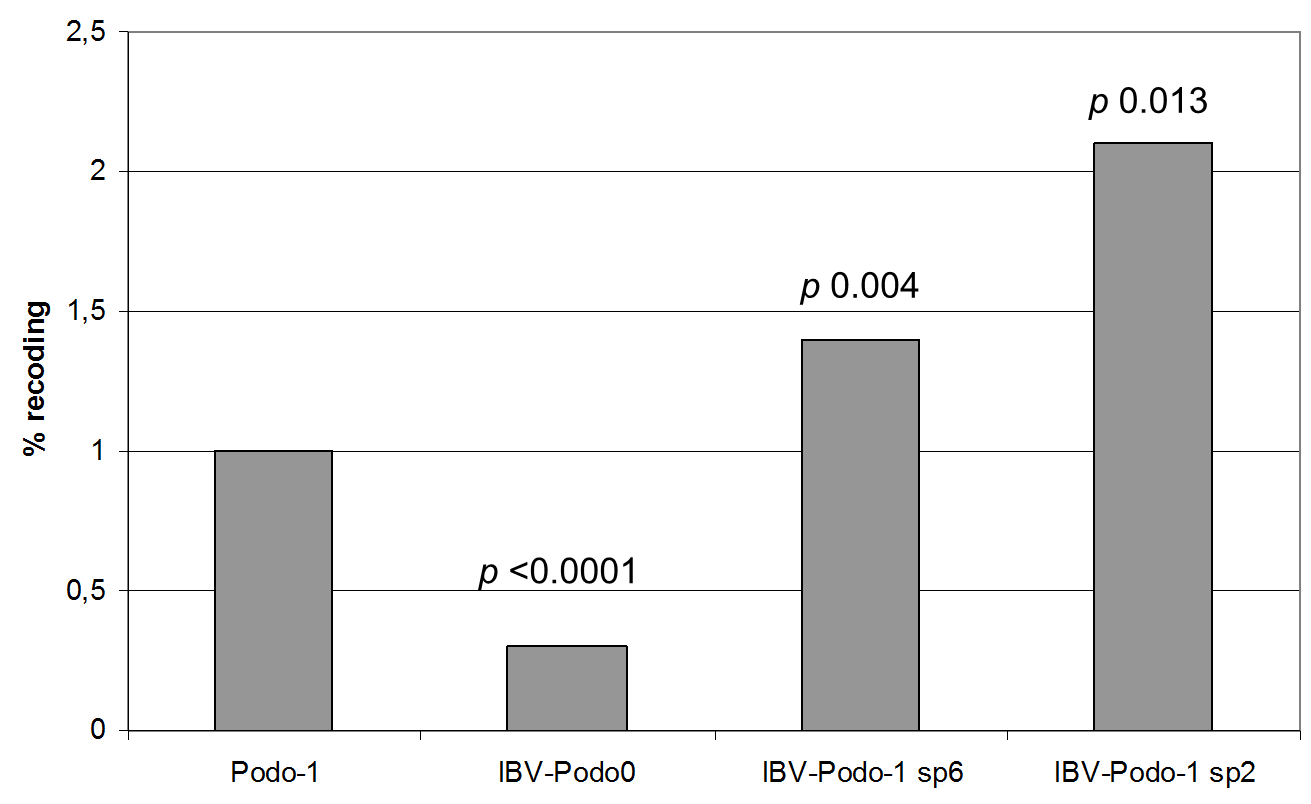

Supplement: Figure S4 — −1 Frameshifting efficiency quantified in S. cerevisiae . The −1 frameshifting efficiency of each Podo and IBV-Podo sequence was quantified as described in Materials and Methods. The p-value with the Podo-1 data is indicated for each sequence tested. (TIF) [file pone.0073772.s004.tif]
